# Editorial: Reimagining human movement: the impact of extended reality on physical and emotional experience

**DOI:** 10.3389/fpsyg.2026.1900819

**Published:** 2026-07-28

**Authors:** André Ramalho, José M. Cancela Carral, Catarina Gonçalves, Pedro Duarte-Mendes

**Affiliations:** 1 Department of Sports and Well-Being, Polytechnic University of Castelo Branco, Castelo Branco, Portugal; 2 Sport Physical Activity and Health Research & Innovation Center (SPRINT), Castelo Branco, Portugal; 3 PSYLAB—Faculty of Human Kinetics, University of Lisbon, Lisbon, Portugal; 4 University of Vigo, Vigo, Spain; 5 Department of Arts, Humanities and Sport, Polytechnic University of Beja, Beja, Portugal; 6 Comprehensive Health Research Centre (CHRC), Evora, Portugal

**Keywords:** affective engagement, embodiment, human movement, immersive technology, psychomotor performance

Extended reality (XR) is no longer a peripheral tool for reproducing movement tasks in digital form. Across virtual, augmented, and mixed reality, it is becoming a way of reorganizing the conditions under which human movement is perceived, learned, controlled, and sustained. The central question, therefore, is not simply whether immersive technologies can increase activity or improve performance, but how they alter the perceptual, affective, cognitive, social, and sensorimotor architectures through which movement becomes possible and meaningful (Ramalho et al., 2024). This Research Topic brings that shift into focus. Read together, its contributions show a field moving beyond novelty and feasibility toward a more mechanistic and context-sensitive science of embodied experience, consistent with recent evidence that body tracking, avatar representation, and immersive interaction can shape psychological and behavioral responses in virtual environments (Bailenson et al., 2025), and with growing attention to the acceptability of XR-based physical activity across populations.

The first axis concerns motivation and adherence. Tan et al. compare exergaming aerobic dance with instructor-led aerobic dance and reveal a productive tension: the traditional format resulted in a higher step rate, whereas exergaming elicited greater enjoyment and intrinsic motivation. The implication is not that gamified exercise is inherently superior, but that movement interventions must distinguish physiological intensity from experiential reward. Ciemer et al. extend this point in older adults through *EXploVR*, a fully immersive fall-prevention exergame that combines natural locomotion with cognitive challenge. Improvements in gait-related outcomes were accompanied by high enjoyment and perceived meaningfulness, indicating that mobility interventions may become more effective when they address confidence, safety, and everyday relevance, not only motor output.

A second line of inquiry concerns learning. Suo et al. show that high-embodiment augmented reality improved retention and transfer in biology instruction while reducing cognitive load, although lower embodiment was perceived as easier to use. The finding captures a design problem at the heart of educational XR: embodied interaction can deepen learning, but only if its complexity does not become a new barrier to access. Li et al. reach a related conclusion in XR-based soccer learning. Game elements did not directly increase learning effort; their effect was transmitted through flow and mental toughness, while self-control acted through both direct and indirect pathways. Immersive learning, in this view, depends less on adding stimulation than on transforming situated experience into durable motivational and self-regulatory resources.

A third set of contributions turns to embodiment itself. Laattala et al. examine dancing with an artificial intelligence partner in virtual and mixed reality and show that stronger presence in virtual reality did not translate into a universal preference. Some participants valued immersion and reduced awareness of their own bodies, while others preferred the safety and spatial orientation afforded by mixed reality. Greater immersion does not necessarily result in a better experience. Kober et al. add neurophysiological precision to this claim. In a virtual button-press task, realistic and skeletal hand models produced comparable neuronal and behavioral responses, whereas, during a more complex virtual object-grasping task, realistic hands elicited stronger activation in motor brain areas. These findings suggest that virtual body design is not merely decorative; under richer and more ecologically valid task demands, it can modulate sensorimotor processing and may have implications for future clinical virtual reality applications.

Several studies widen this perspective beyond XR while remaining essential to its future development. Pan et al. use eye tracking to show that walking, jogging, and cycling in greenway environments organize attention differently, with vegetation, roads, and distant views carrying distinct perceptual and cognitive functions. Movement is thus strongly shaped by environmental characteristics, not merely environmentally located. Guo et al. demonstrate that personality traits and emotional states interact with gait stability, speed, locomotor efficiency, and joint function, opening possibilities for psychologically responsive movement systems and virtual character modeling. Zhang et al. locate embodiment within digital social life, showing that higher social media comparison is associated with lower physical self-efficacy through body shame and body appreciation. These findings matter for XR because future immersive systems will be experienced by individuals already shaped by comparison, affect, identity, and cultural ideals.

Other contributions sharpen the psychological specificity of movement. Gu et al. show that risk perception in mountaineering is linked to re-participation intention through flow experience, with social support strengthening this pathway. Darrin et al. report that long-term *Tai Chi* practice is associated with stronger inhibitory control and working memory, but not cognitive flexibility. Together, these findings resist broad claims about movement as uniformly beneficial. They suggest, instead, that movement practices produce specific effects that depend on task structure, practice history, risk appraisal, attention, social context, and the meanings participants attach to continued engagement.

The Research Topic is also anchored by conceptual and methodological contributions. Bös and Mechling argue for a psychomotor framework that moves beyond narrow accounts of motor performance toward an action-oriented, biocultural, and ability-based understanding of human behavior. Their model clarifies why movement cannot be reduced to biomechanical output: it is intentional action embedded in cognitive, emotional, developmental, and social conditions. Robotham et al. address the corresponding evaluation problem by presenting the Quality and Experience Evaluation Tool for interactive virtual reality, integrating perceptual, cognitive, behavioral, and interactivity data. As XR systems become more adaptive and temporally coupled with users, evaluation must account for the interaction process itself as an integral component of the user experience.

**Figure 1:**
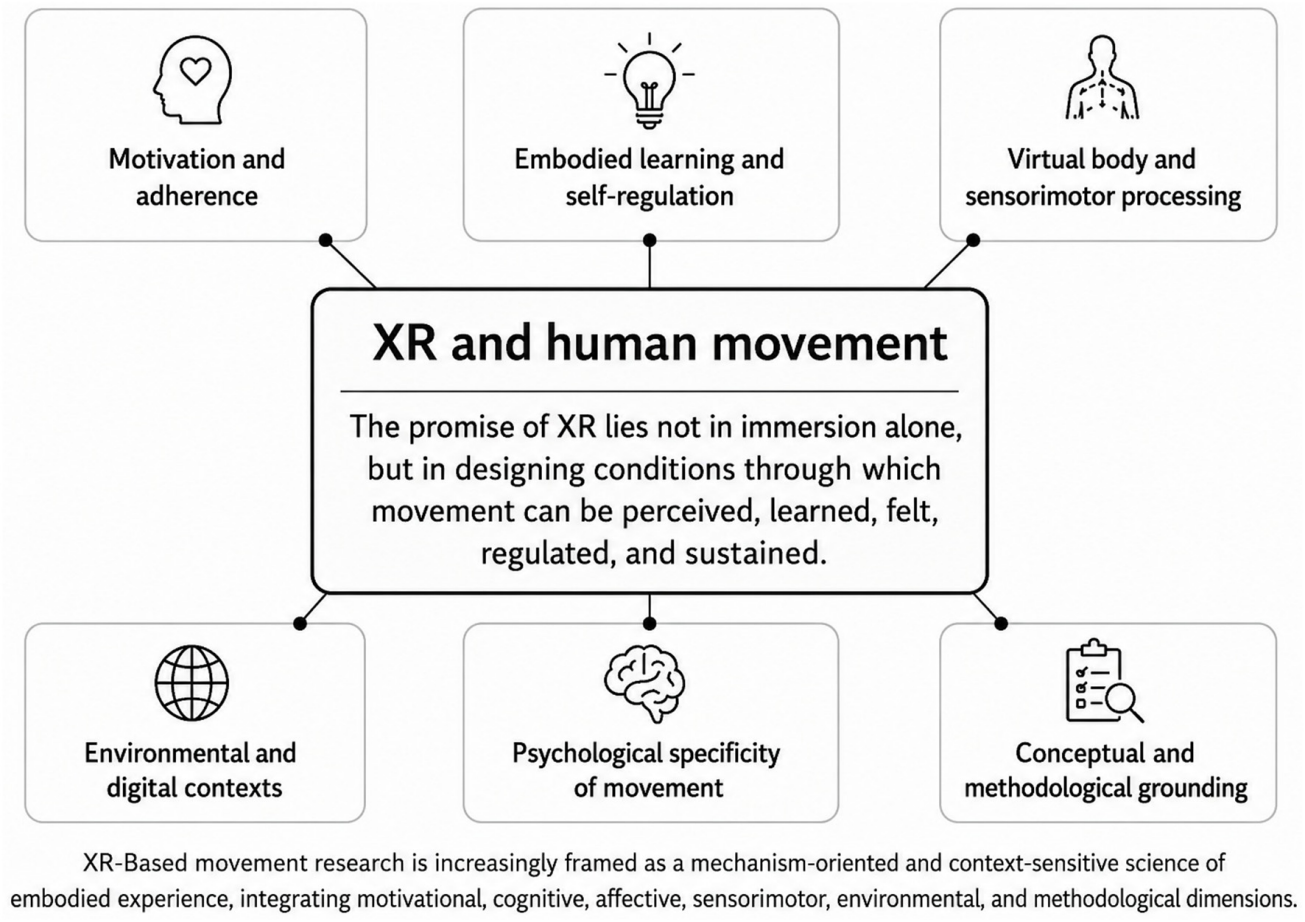
Conceptual synthesis of the Research Topic.

Read as a whole, this Research Topic (see Figure 1) suggests that the promise of XR in human movement does not lie in immersion alone. It lies in the possibility of designing environments in which perception, affect, cognition, social meaning, bodily representation, and motor performance can be studied as closely interconnected. The field is maturing, but the evidence also calls for restraint: several findings remain based on pilot studies, cross-sectional, short-term, or context-specific. Future work will need stronger longitudinal designs, clearer comparative frameworks, and greater attention to accessibility, transfer, and equity. The most productive path is neither technological enthusiasm nor technological skepticism, but a more discriminating science of embodied experience: one able to ask what XR enables, for whom, under which conditions, through which mechanisms, and with what consequences for the ways humans learn, move, feel, and persist.
